# Potential association among posterior fossa bony volume and crowdedness, tonsillar hernia, syringomyelia, and CSF dynamics at the craniocervical junction in Chiari malformation type I

**DOI:** 10.3389/fneur.2023.1069861

**Published:** 2023-02-20

**Authors:** Shengxi Wang, Dongao Zhang, Kun Wu, Wayne Fan, Tao Fan

**Affiliations:** ^1^Department of Spinal Spine Surgery, Sanbo Brain Hospital, Capital Medical University, Beijing, China; ^2^Faculty of Science, University of British Columbia, Vancouver, BC, Canada

**Keywords:** Chiari malformation type I, posterior cranial fossa, cerebellar tonsil, syringomyelia, CSF dynamics

## Abstract

**Objective:**

The characteristic morphological parameters (bony posterior fossa volume (bony-PFV), posterior fossa crowdness, cerebellar tonsillar hernia, and syringomyelia) and CSF dynamics parameters at the craniocervical junction (CVJ) in Chiari malformation type I (CMI) were measured. The potential association between these characteristic morphologies and CSF dynamics at CVJ was analyzed.

**Methods:**

A total of 46 cases of control subjects and 48 patients with CMI underwent computed tomography and phase-contrast magnetic resonance imaging. Seven morphovolumetric measures and four CSF dynamics at CVJ measures were performed. The CMI cohort was further divided into “syringomyelia” and “non-syringomyelia” subgroups. All the measured parameters were analyzed by the Pearson correlation.

**Results:**

Compared with the control, the posterior cranial fossa (PCF) area, bony-PFV, and CSF net flow were significantly smaller (*P* < 0.001) in the CMI group. Otherwise, the PCF crowdedness index (PCF CI, *P* < 0.001) and the peak velocity of CSF (*P* < 0.05) were significantly larger in the CMI cohort. The mean velocity (MV) was faster in patients with CMI with syringomyelia (*P* < 0.05). In the correlation analysis, the degree of cerebellar tonsillar hernia was correlated with PCF CI (*R* = 0.319, *P* < 0.05), MV (*R* = −0.303, *P* < 0.05), and the net flow of CSF (*R* = −0.300, *P* < 0.05). The Vaquero index was well correlated with the bony-PFV (*R*= −0.384, *P* < 0.05), MV (*R* = 0.326, *P* < 0.05), and the net flow of CSF (*R* = 0.505, *P* < 0.05).

**Conclusion:**

The bony-PFV in patients with CMI was smaller, and the MV was faster in CMI with syringomyelia. Cerebellar subtonsillar hernia and syringomyelia are independent indicators for evaluating CMI. Subcerebellar tonsillar hernia was associated with PCF crowdedness, MV, and the net flow of CSF at CVJ, while syringomyelia was associated with bony-PFV, MV, and the net flow of CSF at the CVJ. Thus, the bony-PFV, PCF crowdedness, and the degree of CSF patency should also be one of the indicators of CMI evaluation.

## 1. Introduction

Chiari malformation type I (CMI) is diagnosed when the cerebellar tonsil exceeds the foramen magnum (FM) by 5 mm on magnetic resonance imaging (MRI) (1, 2). This definition is concise, but it can mislead researchers to overlook the complex nature of this multifactorial congenital developmental malformation (3). In recent years, many studies have found that the incidence of CMI is usually accompanied by significant morphological (2, 4, 5) and posterior cranial fossa (PCF) volume changes (4). Some characteristic changes can exist independently or correlate with each other, and cerebellar tonsillar hernia may no longer be the sole evaluation criterion for CMI (6). In addition, numerous studies have indicated that bony dysplasia of PCF underlies the pathology of patients with CMI; however, most studies have not clearly measured the changes in bony posterior fossa volume (bony-PFV) (7, 8), and few studies have further analyzed the relationship between the characteristic changes in bony-PFV and cerebrospinal fluid (CSF) circulation at the craniocervical junction (CVJ).

Phase-contrast magnetic resonance imaging (PC-MRI) has been rigorously developed, and the study of CSF dynamics is no longer limited to the anatomical perspective but to recognize and study the CMI disease dynamically, but the exploration of CSF dynamics is still unclear. We often observe patients with CMI with mild cerebellar tonsillar hernia (9), and some scholars believe that the differences in symptom types and severity of CMI may be attributed to variability in CSF dynamics, rather than the degree of herniation of the cerebellar tonsil (10). Based on different regions of interest, increasing (11–14) or decreasing (15) CSF velocity has been previously reported. Ibrahimy et al. also simulated CSF motion to measure integrated longitudinal impedance (16).

There are few reports about the correlation between the morphological changes in CMI and CSF dynamics (15, 17), and the quantification of CSF dynamics remains highly controversial. Therefore, different from previous studies, we use computed tomography (CT) data of bony-PFV in this study to investigate the potential association among the PCF bony volume and crowding, cerebellar tonsillar hernia, syringomyelia (SM), and the dynamic changes in CSF at the CVJ with CMI and to evaluate indicators for predicting the patency of CSF.

## 2. Materials and methods

### 2.1. Subjects

Approval for this study was obtained from our institutional review board, and consent was obtained from all subjects. Seventy-three hospitalized and surgically treated adult patients were retrospectively evaluated from January 2019 to January 2022, and hospitalized adults diagnosed with unruptured intracranial aneurysms matched for sex and age served as the control group. All enrolled subjects underwent thin-layer CT and PC-MRI examinations of the neck. All data were measured preoperatively.

The inclusion criteria were as follows:

- Primary diagnosis as CMI (1): the cerebellar tonsil exceeds the FM by 5 mm on MRI.
- Age over 16 years.

The exclusion criteria were as follows:

- Basilar invagination (BI) (18), atlantoaxial dislocation (AAD) (19), Klippel–Feil anomaly (20), or any other bony malformations in the CVJ, such as rheumatoid arthritis, with the pB-C2 line (21) more than 9 mm.
- Acquired CMI or spinal cord malformation, such as hydrocephalus, intracranial neoplasm, or tethered spinal cord.
- A history of skull or spinal trauma or surgery.

### 2.2. Radiologic evaluation

Thin-slice CT imaging was performed on a 128-slice multidetector scanner (Siemens, Germany) with a thickness of 0.5 mm. As described previously (4), the bony-PFV was modeled using Mimics software (version 18.0.0.525, Materialize NV, Leuven, Belgium). After DICOM CT data were imported, the 3D coordinate system was readjusted, and a mask was established with a threshold set between 850 and 1,250 Hu. The range of the mask is defined as a space defined by a series of skeletal anatomical structures, and the volume is automatically calculated.

Phase-contrast magnetic resonance imaging was performed using a 1.5T scanner (Philips, Netherlands). The hydrodynamics of CSF measured during a cardiac cycle consisted of 36 phase and amplitude maps. The coding speed was 12 cm/s, the thickness was 4 mm, the reverse corner was 15°, and the direction of speed coding was cranial to caudal.

### 2.3. Measurement parameters

(1) Tonsil descent: the distance between the lowermost margin of the cerebellar tonsils and McRae's line.
(2) Syrinx length: the number of segments that syrinx span the spinal cord.
(3) VI (22): Vaquero index, the ratio of the greatest diameter of the syrinx to that of the spinal cord.
(4) HB area: hindbrain area, the area of the cerebellum and the brainstem on a midsagittal MRI image, excluding herniated tonsils.
(5) PCF area: posterior cranial fossa area, the area bordered by the clivus, the tentorium, the occipital bone, and the FM on MRI in the midsagittal view.
(6) PCF CI: PCF crowding index, calculated as HB/PCF × 100%.
(7) Bony-PFV (4): defined as the space between the saddleback, the bilateral petrous bone, the intraoccipital protuberance, and the FM.
(8) PV_max_: maximum positive velocity, the maximum cranial velocity of CSF at FM in a cardiac cycle.
(9) NV_max_: maximum negative velocity, the maximum caudal velocity of CSF at FM in a cardiac cycle.
(10) AbsMV: the absolute value of the mean velocity.
(11) Net flow: the net CSF flow at FM during a cardiac cycle.

**Figure 1:**
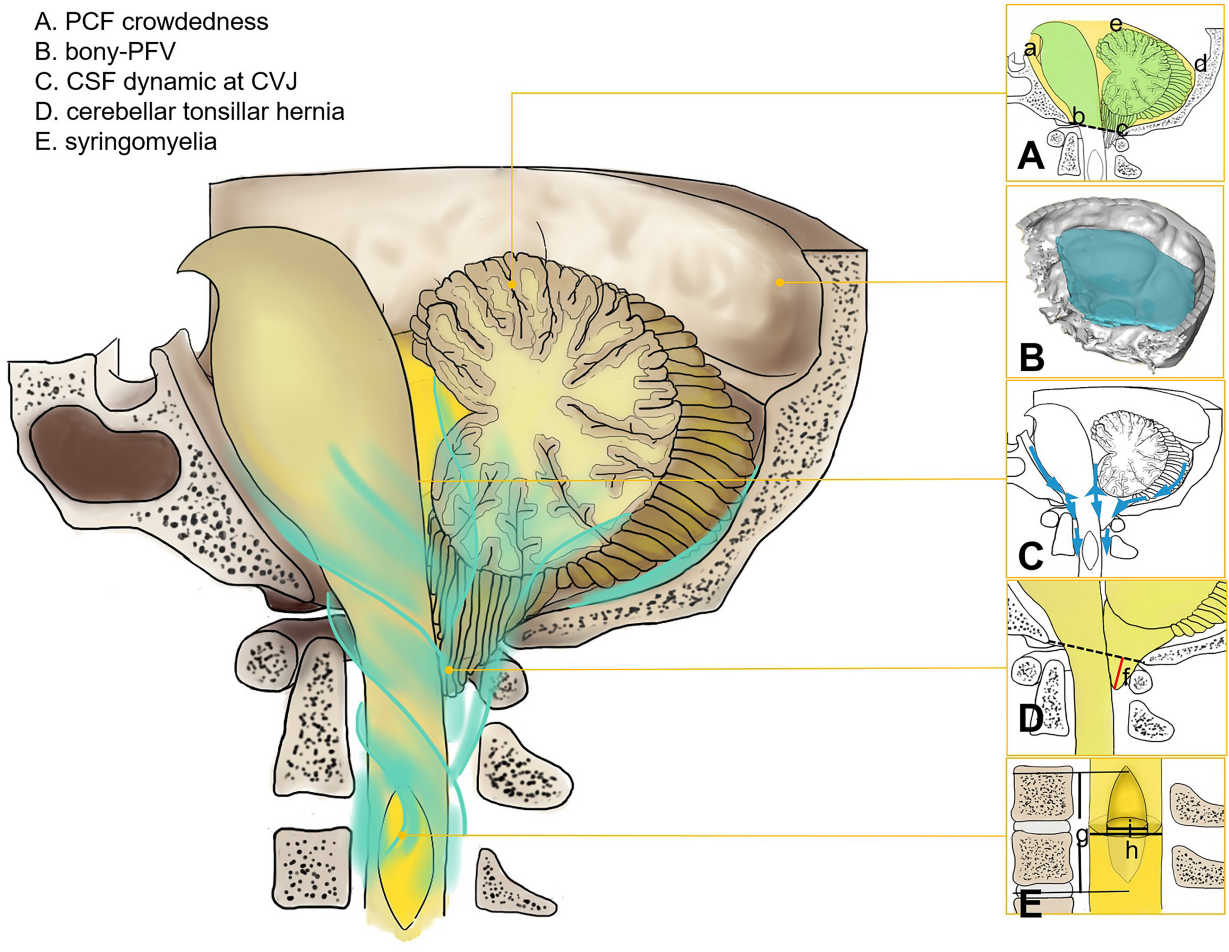
Morphology and physiological parameters of CSF at CVJ in Chiari malformation I. **(A)** PCF crowdedness, McRae's line (b–c), and tentorium cerebelli (d–e). The hindbrain area is represented in green, and the PCF area is shown in yellow (a–e). **(B)** Three-dimensional model of PFV. **(C)** CSF circulation at CVJ. **(D)** Cerebellar tonsil descent (f). **(E)** syrinx length (g), maximum diameter of syrinx (i), and spinal cord diameter (h).

**Figure 2:**
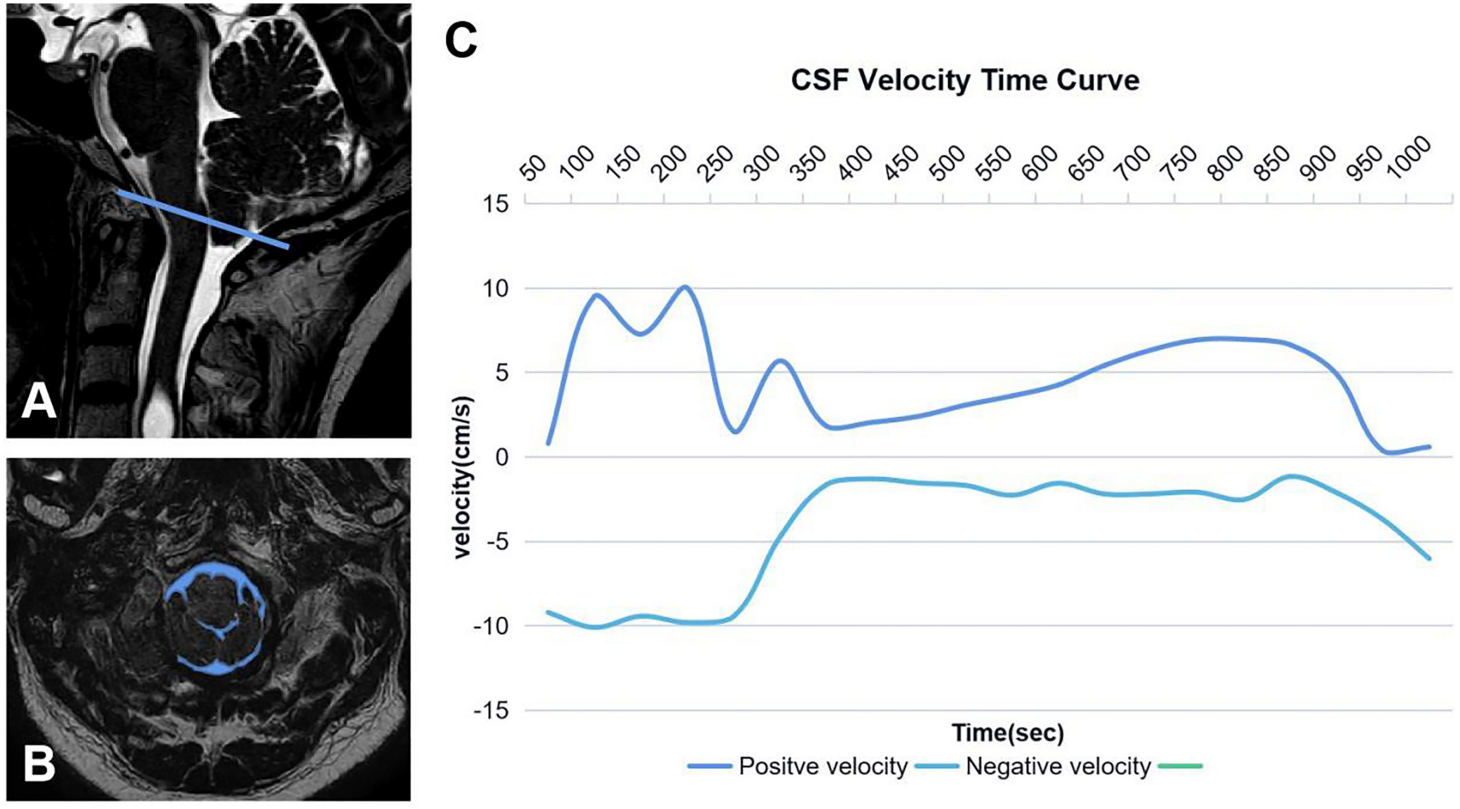
Region of interest of CSF **(A, B)**. The blue line represents McRae's line, and the blue area is the cross-sectional area of CSF at CVJ. CSF time–velocity curve in one cardiac cycle **(C)**.

Test–retest reliability was repeatedly measured within 2 weeks by two neurosurgery residents (S.X. and Y.B.R.), and the average value of the two groups was recorded and analyzed (Figures 1, 2).

### 2.4. Statistical analysis

The conformance test of the two measures was performed using intragroup correlation coefficients, measured as ($\bar{x}\pm s$). An independent sample *t*-test was used to determine the significance of the measures between control groups and patients with CMI between CMI with and without SM. The chi-square test compared count data between the two groups. The Pearson correlation analysis was used to investigate the morphological and physiological indices. Statistical analyses were performed using IBM SPSS software version 25.0 (SPSS Inc., Chicago, Illinois, USA). Significance was indicated by a *P* < 0.05. For the multiple corrections, the false discovery rate (FDR) was used based on the Benjamini–Hochberg procedure (23).

## 3. Results

A total of 48 patients (40 female subjects, 43.5 ± 2.24 years) in the CMI group and 46 control subjects (36 female subjects, 45.0 ± 1.22 years) were included in this study. There was no difference in age (*P* = 0.594) or sex (*P* = 0.137). The CMI group presented with cough-related occipital headache (54.2%), limb numbness (50%), neuropathic pain (22.9%), sensory disturbances (20.8%), muscle weakness (25%), amyotrophy (2%), and choking or hoarseness on drinking water (2%).

### 3.1. Comparison of parameters between the control group and the CMI group

A total of 10 out of the 11 morphological and physiological parameters were significantly different between the control group and the CMI cohort (Table 1). The parameters of the two groups were tested for consistency, and the lowest intragroup correlation coefficient (ICC) value was 0.896, which indicates good consistency.

**Table 1:** Observational characteristics of the study subjects.

**Variables**	**Control (*n* = 46)**	**CMI (*n* = 48)**	***p***	**CMI**		
				**Non-SM (*****N*** = **5)**	**SM (*****N*** = **43)**	***P***
Age (y)	45.0 ± 1.22	43.5 ± 2.24	0.594	42.4 ± 6.99	43.7 ± 2.44	0.858
Tonsil descent (mm)	-	9.85 ± 0.74	-	9.49 ± 0.76	12.6 ± 2.7	0.186
Syrinx length (number)	-	7.03 ± 0.46	-	-	6.88 ± 0.43	-
VI (%)	-	64.79 ± 3.25	-	-	64.24 ± 2.78	-
HB area (mm^2^)	2,101.32 ± 54.08	2,432.23 ± 45.52	0.066	2,380.38 ± 246.28	2,438.88 ± 42.62	0.688
PCF area (mm^2^)	3,476.39 ± 62.73	3,088.44 ± 41.45	<0.001	3,032.62 ± 165.03	3,095.59 ± 42.59	0.635
PCF CI (%)	66.16 ± 0.87	78.64 ± 0.80	<0.001	77.78 ± 5.19	78.75 ± 0.72	0.790
bony-PFV (ml)	158.01 ± 3.35	129.46 ± 4.84	<0.001	134.77 ± 3.39	127.36 ± 4.87	0.316
PV_max_ (cm/s)	8.29 ± 0.39	9.87 ± 0.51	0.016	9.14 ± 0.18	9.82 ± 0.57	0.567
NV_max_ (cm/s)	−7.67 ± 0.70	−10.05 ± 0.48	0.014	−9.80 ± 0.52	−9.90 ± 0.53	0.929
absMV (cm/s)	1.01 ± 0.16	0.77 ± 0.13	0.254	0.18 ± 0.12	0.86 ± 0.17	0.039
Net flow (ml)	4.05 ± 0.64	1.01 ± 0.28	0.001	0.36 ± 0.19	1.12 ± 0.30	0.209

In the CMI group, the cerebellar tonsillar hernia was (9.85 ± 0.74) mm, and the syrinx was across the level of the spinal cord (7.03 ± 0.46), VI was (64.79 ± 3.25). Compared with the control group, the PCF area (*P* < 0.001), bony-PFV (*P* < 0.001), and net flow (*P* < 0.001) were significantly smaller in the CMI cohort. The PCF CI (*P* < 0.001), PV_max_ (*P* < 0.05), and NV_max_ (*P* < 0.05) in the CMI cohort were higher than those in the control group. There were no significant differences in the HB area (*P* = 0.066) or absMV (*P* = 0.254) between the two groups.

### 3.2. Comparison of parameters between patients with CMI with and without syringomyelia

A total of 43 patients (89.6%) were complicated with SM in the enrolled CMI cohort (Table 1). Compared with the non-SM group, the absMV (*P* < 0.05) was significantly higher in the SM group. Bony-PFV was smaller in the SM group (127.36 ± 4.87 ml) than in the non-SM group (134.77 ± 3.39 ml) though the difference was not statistically significant (*P* = 0.316).

### 3.3. Correlation among the PCF bony volume and crowdedness, cerebellar tonsillar hernia, syringomyelia, and CSF dynamic measures in the CMI cohort

**Figure 3:**
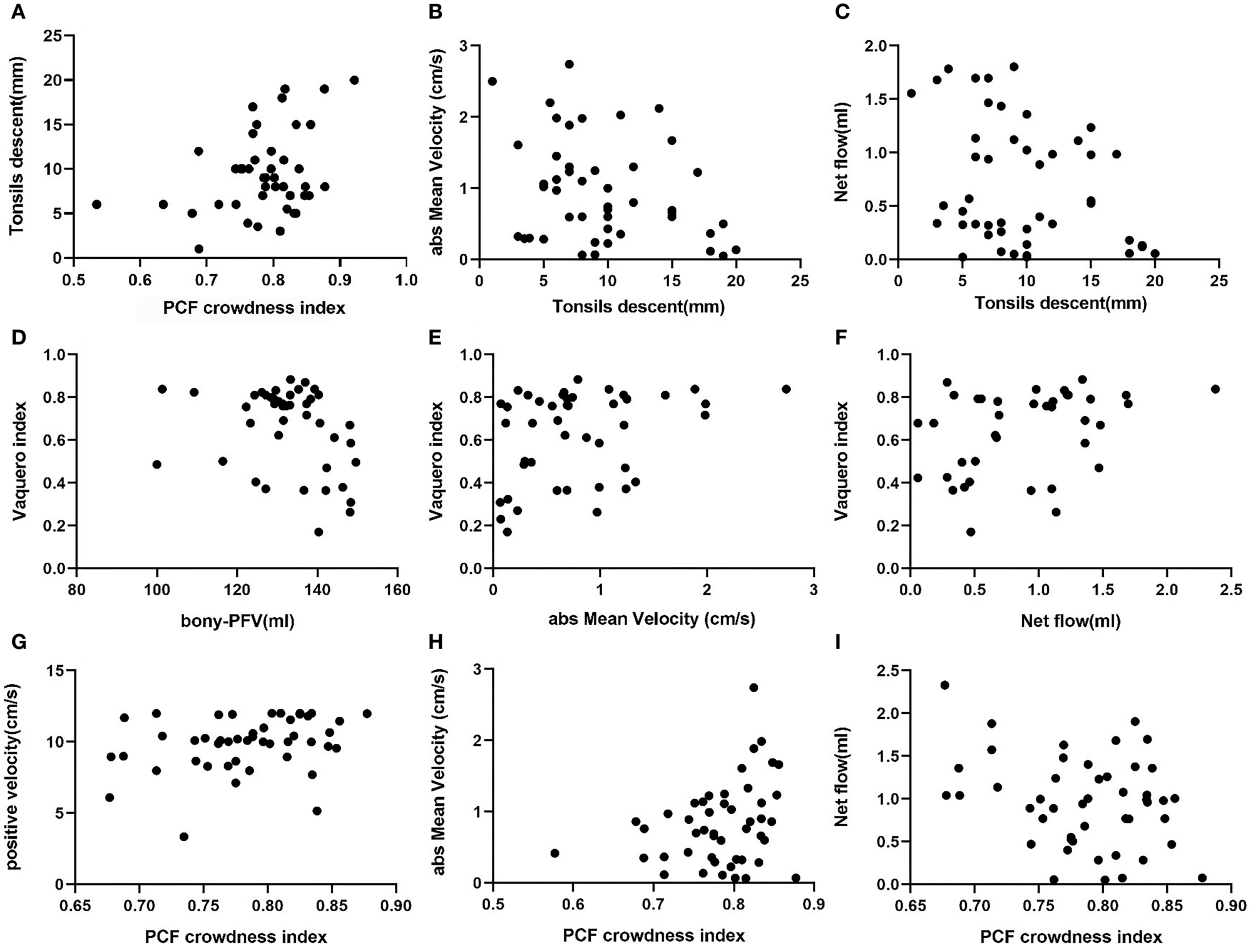
Scatter plots of correlations between characteristic morphological and physiological measurements of CMI: **(A)** PCF CI associated with tonsillar descent, **(B)** tonsillar descent associated with absMV, **(C)** tonsillar descent associated with CSF net flow, **(D)** bony-PFV associated with, **(E)** absMV associated with VI, **(F)** CSF net flow associated with VI, **(G)** PCF CI associated with PVmax, **(H)** PCF CI associated with absMV, and **(I)** PCF CI associated with CSF net flow.

Pearson correlation coefficients and P-values are listed in Table 2. The scatter plots of correlations between characteristic morphological and physiological measurements of the CMI cohort are shown in Figure 3. In general, most of the morphological and physiological measurements were weakly correlated in dependence tests, and only a few were moderately correlated.

**Table 2:** Pearson correlation between every two parameters in the CMI group.

**Correlation**		**Cerebellar tonsillar hernia**	**Syrinx expansion**		**PCF**		**CSF dynamic**		
		**Tonsils descent (mm)**	**Syrinx length**	**VI**	**PCF CI**	**Bony-PFV (ml)**	**PV**_max_ **(cm/s)**	**NV**_max_ **(cm/s)**	**absMV (cm/s)**
Syrinx expansion	Syrinx length	*r* = 0.029; *P* = 0.863							
	VI	*r* = 0.007; *P* = 0.966	*r* = 0.575; *P* = 0.000^***^						
PCF	PCF CI	*r* = 0.319^A^; *P* = 0.027^*^	*R* = 0.215; *P* = 0.209	*r* = −0.157; *P* = 0.328					
	Bony-PFV (ml)	*r* = −0.014; *P* = 0.960	*r* = 0.270; *P* = 0.372	*r* = −0.384^D^; *P* = 0.021^*^	*r* = −0.468; *P* = 0.001^**^				
CSF dynamic	PV_max_ (cm/s)	*r* = 0.036; *P* = 0.796	*r* = 0.184; *P* = 0.424	*r* = −0.169; *P* = 0.411	*r* = 0.295^G^; *P* = 0.042^*^	*r* = −0.301; *P* = 0.186			
	NV_max_ (cm/s)	*r* = 0.051; *P* = 0.809	*r* = −0.032; *P* = 0.892	*r* = 0.247; *P* = 0.280	*r* = 0.076; *P* = 0.724	*r* = 0.253; *P* = 0.268	*r* = −0.860; *P* = 0.000^***^		
	absMV (cm/s)	*r* = −0.303^B^; *P* = 0.036^*^	*r* = 0.159; *P* = 0.315	*r* = 0.326^E^; *P* = 0.031^*^	*r* = 0.289^H^; *P* = 0.046^*^	*r* = 0.199; *P* = 0.414	*r* = 0.511; *P* = 0.011^*^	*r* = −0.337; *P* = 0.108	
	Net flow (ml)	*r* = −0.300^C^; *P* = 0.038^*^	*r* = 0.055; *P* = 0.823	*r* = 0.505^F^; *P* = 0.046^*^	*r* = −0.292^I^; *P* = 0.044^*^	*r* = 0.105; *P* = 0.787	*r* = 0.023; *P* = 0.923	*r* = 0.624; *P* = 0.7938	*r* = 0.9256; *P* = 0.000^***^

A Cerebellar tonsillar hernia associated with PCF CI,

B cerebellar tonsillar hernia associated with absMV,

C cerebellar tonsillar hernia associated with CSF net flow,

D bony-PFV associated with VI.

E absMV associated with VI,

F CSF net flow associated with VI,

G PVmax associated with PCF CI,

H absMV associated with PCF CI,

and

I PCF CI associated with CSF net flow.

* *P* < 0.05;

** *P* < 0.01;

*** *P* < 0.001.

#### 3.3.1. PCF bony volume and crowding

The indicators related to PCF are represented by PCF CI and bony-PFV. There was a significant correlation between bony-PFV and PCF CI (*R* = −0.468, *P* < 0.01). The correlation that reached a moderate level was between bony-PFV and VI (*R* = −0.384, *P* < 0.05). Similarly, PCF CI was also weakly correlated with tonsil descent (*R* = 0.319, *P* < 0.05), PV_max_ (*R* = 0.295, *P* < 0.05), absMV (*R* = 0.289, *P* < 0.05), and net flow (*R* = −0.292, *P* < 0.05). However, neither tonsil descent (*R* = −0.014, *P* = 0.960) nor the degree of patency of CSF was significantly associated with bony-PFV.

#### 3.3.2. Cerebellar tonsil herniation parameters

In addition to PCF CI, tonsil descent was slightly associated with absMV (*R* = −0.303, *P* < 0.05) and net flow (*R*= −0.300, *P* < 0.05), while there was no correlation with syrinx expansion.

#### 3.3.3. Syringomyelia parameters

The degree of syringomyelia was measured by syrinx length and VI. The results showed that VI was slightly correlated with bony-PFV (*R*= −0.384, *P* < 0.05) and absMV (*R* = 0.326, *P* < 0.05) and moderately correlated with CSF net flow (*R* = 0.505, *P* < 0.05). However, the degree of syrinx expansion was not correlated with the degree of tonsil descent.

#### 3.3.4. CSF dynamic parameters

In this analysis, PV_max_, NV_max_, absMV, and CSF net flow were used as reference indicators for CSF patency in the FM. The results showed that PV_max_ was correlated with PCF CI (*R* = 0.295, *P* < 0.05). Similarly, the absMV was correlated with tonsil descent (*R* = −0.303, *P* < 0.05), VI (*R* = 0.326, *P* < 0.05), and PCF CI (*R* = 0.289, *P* < 0.05). In addition, CSF net flow was correlated with tonsil descent (*R* = −0.300, *P* < 0.05), VI (*R* = 0.505, *P* < 0.05), and PCF CI (*R* = 0.292, *P* < 0.05).

## 4. Discussion

The pathogenesis of CMI remains unclear, and a theory of bone dysplasia has been proposed since the malformation of PFV cannot match the normal development of the posterior fossa nerve tissue, resulting in cerebellar tonsillar hernia down into the spinal canal, and disordered circulation of CSF at CVJ. However, this theory cannot fully explain the pathology of CMI. The data from different studies are inconsistent; some studies have shown a smaller PCF in adults with CMI (15, 24–28), while other studies have reported no difference in PCF between CMI and the general population (29–31). In addition, it should be noted that CT is more accurate in showing the bone structure.

In previous studies, we described the underdeveloped clivus and occipital bone as well as a reduction of the volume of bony PCF in adults with CMI (4). We also first divided CMI into three different degrees of CSF circulation disorders in the anterior, middle, and posterior spaces (32). Here, we quantitatively analyzed the CSF dynamics at CVJ. Different from previous research studies, we used the CT data of bony-PFV to evaluate the relationships of changes in CSF dynamics at CVJ and prominent morphological features (bony-PFV, PCF crowdedness, cerebellar tonsillar hernia, syrinx expansion, and length) in order to investigate the independence and complementarity of these parameters. Finally, we identified that the morphological and physiological indicators are most closely related to the patency of CSF at CVJ.

Pearson correlation analysis was performed for morphological and physiological measures, and a *P* < 0.05 was considered significant. The FDR correction was for reference on account of the limited sample size.

### 4.1. Measurement of characteristic morphological and physiological parameters of CMI

In our study, thin-slice CT data were used to reconstruct the bony PCF (controls −158.01 ± 3.35 ml vs. patients −129.46 ± 4.84 ml, *P* < 0.001). In addition, MRI sagittal images were used to measure PCF crowdedness, which is well correlated with bony-PFV (*R* = −0.468, *P* < 0.001), and linear PCF measurement is a supplement to volumetric measurement (15). We found no difference in the hindbrain area between control subjects and patients with CMI, and significant differences in PCF CI (controls −66.16 ± 0.87% vs. patients −78.64 ± 0.87%, *P* < 0.001), which demonstrates that the PFV was disproportionate to its contents and suggests that a better feature of CMI is the bony volume and crowdedness of the PCF, which is consistent with previous reports (15).

A meta-analysis showed that the peak velocity of CSF was most often reported at the FM-C1 vertebral level (33). In this section, we compared and quantified the CSF flow in the FM region during the cardiac cycle between control subjects and patients with CMI. It was found that the peak velocity of the patients with CMI was higher than that of the controls regardless of the direction of CSF flow. The absolute value was (9.87 ± 0.51) cm/s, which was similar to the results of Bunck et al. (12), Yiallourou et al. (13), and Iskandar et al. (14) but different from Alperin et al. (15). According to Bernoulli's principle, the flow velocity of CSF through the FM increases and the fluid pressure decreases, suggesting that the tendency of cerebellar tonsils and other posterior brain tissues to herniate into the spinal canal increases, which worsens the CSF circulation disturbance at CVJ. More significantly, patients in the CMI cohort had significantly less CSF flow at FM than the control group regardless of whether there was syringomyelia. This is consistent with Alexander's results (12), but there was no significant difference in the absMV of CSF between control subjects and patients with CMI, which may be due to the overcrowding of the FM, increased CSF turbulence and flow disorder, and the lateral fluid offset part of the energy along the flow direction. Indeed, some studies have simulated the trend of CSF turbulence at FM (11, 12).

Approximately 89.6% of patients with CMI had syringomyelia, which was similar to previous reports (34). When the CMI cohort was grouped according to whether there was syringomyelia, the descending movement of the cerebellar tonsils in the SM-CMI group seemed to be more obvious (SM 12.6 ± 2.7 mm vs. non-SM 9.49 ± 0.76 mm, *P* = 0.186), but there was no significant difference, and there was no difference in the bony-PFV, PCF CI, CSF peak velocity, or net flow at CVJ. The most notable finding is that the absMV in the CMI-SM group was higher than that in CMI without SM. This illustrates that the formation of syringomyelia has a close relationship with CSF circulation, and the examination of CSF dynamics is critically important. Changes in CSF circulation can be used to prospectively predict the direction of disease progression.

### 4.2. Correlation analysis among bony-PFV, PCF crowdedness, cerebellar tonsillar hernia, syringomyelia, and CSF dynamics at CVJ

Previous studies have been limited to correlation analysis between PCF size and CSF flow (15). In our study, we not only considered the changes in the bony structure of the PCF but also included soft evaluation parameters such as PCF crowding, cerebellar tonsillar ectopia, the degree of syrinx dilation, and CSF flow at CVJ to conduct a comprehensive correlation analysis.

Among the parameters of PCF, the VI is the only parameter that is correlated with the bony-PFV, which suggests that patients with CMI whose bony-PFV are small are more prone to syringomyelia in the course of the disease. If abnormal narrowness of bony-PFV is found in the early stage of clinical diagnosis and treatment, close follow-up and vigilance should be increased, and intervention should be implemented in the early stage of syringomyelia progression. Interestingly, the severity of cerebellar tonsillar hernia was disproportionate to the bony-PFV. Therefore, we further measured the PCF CI and found that it was potentially correlated with cerebellar tonsillar hernia and PV_max_ of CSF. This confirmed that the congestion of the PCF may be one of the factors contributing to the downward herniation of the cerebellar tonsils into the spinal canal. The prolapse of the cerebellar tonsils exacerbates the stenosis of CVJ, and the CSF circulation is blocked.

Among the relevant parameters of syringomyelia, the syrinx length seems to have no correlation with any of the parameters measured here. The VI was not only related to the bony-PFV but also related to the velocity of CSF, and even the net flow of CSF reached a moderate degree of correlation. This result suggests that the alteration of the high dynamic CSF flow of CMI may play an important role in the pathogenesis of syringomyelia.

In the correlation analysis of cerebellar tonsillar hernia, PCF crowdedness, absMV, net flow of CSF, and tonsillar hernia all reached a relevant level. This result suggests that the cerebellar tonsillar hernia in patients with CMI may be the result of the combined effect of PCF crowding and CSF changes. This is, indeed, clinically true; however, some patients have a “spacious” PCF, but severe cerebellar tonsillar hernia can still be observed. This may be related to CSF circulation disorder, and the choice of future treatment strategy should take the PCF bony volume, crowdedness, and CSF circulation factors into consideration. In addition, we noted that cerebellar tonsillar hernia was negatively correlated with absMV. We hypothesized that the more severe the cerebellar tonsillar hernia, the more crowded the CVJ, and the decrease in net flow, and the CSF flow becomes more disrupted and the lateral fluid offsets some of the energy along the flow direction, thus showing a decrease in mean flow velocity.

Among the hydrodynamic parameters of CSF at FM, as mentioned earlier, the absMV and net flow were weakly correlated with the degree of PCF CI, cerebellar tonsillar hernia, and VI. Lower herniated cerebellar tonsils and crowded PCF are often accompanied by faster CSF velocity and less CSF net flow, while high hydrodynamic velocity and high flow of CSF are closely related to syringomyelia formation.

## 5. Conclusion

The bony-PFV in patients with CMI was smaller, and the MV was faster in CMI with syringomyelia. Cerebellar subtonsillar hernia and syringomyelia are independent indicators for evaluating CMI. Subcerebellar tonsillar hernia was associated with PCF crowdedness, MV, and the net flow of CSF at CVJ, while syringomyelia was associated with bony-PFV, MV, and the net flow of CSF at CVJ.

The bony-PFV, PCF crowdedness, and the degree of CSF patency should be considered as combined indexes in the evaluation of patients with CMI.

## 6. Clinical relevance

This study provides some clues for the clinical evaluation and treatment of patients with CMI by quantifying the changes in bony-PFV, PCF crowding, cerebellar tonsillar hernia, syringomyelia, and CSF circulation at CVJ.

1) Cerebellar tonsillar hernia in patients with CMI may be the result of the combined action of PCF and CSF changes. If only the bony-PFV decreased or the PCF was crowded and the CSF PC-MRI examination did not detect an increase in CSF velocity or a decrease in net flow at CVJ, the diagnosis and treatment plan would be mainly to increase the PCF space with bone decompression.
2) If the bony-PFV or PCF CI is within the normal range but the CSF cine indicates increased CSF velocity or decreased net flow at CVJ, subdural decompression or dilated duralplasty may be further performed to improve CSF circulation.
3) If the patient not only has a narrow PCF but also has CSF circulation disorder, the diagnosis and treatment plan should also take into account the expansion of bony-PFV and the clearing of CSF circulation disorder. On the basis of subarachnoid decompression, a part of the cerebellar tonsils can be removed under the pia soft membrane for more complete decompression.

## 7. Limitation

There are several limitations of this study that need to be considered when analyzing the results. First, due to the limited number of samples, this study failed to analyze the possible influence of race, sex, and other factors on these parameters. Second, this study did not include asymptomatic patients or patients with cerebellar tonsillar hernias smaller than 5 mm, which should be investigated in future studies. Finally, this study provides a new direction for thinking about surgical strategies through correlation analysis of the parameters. The realization of this goal requires a large number of patients to receive surgical treatment and long-term follow-up during the study period.

## Data availability statement

The original contributions presented in the study are included in the article/supplementary material, further inquiries can be directed to the corresponding author.

## Ethics statement

The studies involving human participants were reviewed and approved by Ethics Committee of Beijing Sanbo Brain Hospital. The patients/participants provided their written informed consent to participate in this study.

## Author contributions

Conception and design: SW and TF. Acquisition of data: SW and KW. Drafting the article and approved the final version of the manuscript on behalf of all authors: SW. Statistical analysis: SW, DZ, and KW. Study supervision: TF. Analysis and interpretation of data, critically revising the article, and reviewed submitted version of manuscript: All authors. All authors contributed to the article and approved the submitted version.
